# Animal-based welfare indicators of 4 slow-growing broiler genotypes for the approval in an animal welfare label program

**DOI:** 10.3382/ps/pez023

**Published:** 2019-01-23

**Authors:** Helen Louton, Christiane Keppler, Michael Erhard, Otto van Tuijl, Josef Bachmeier, Klaus Damme, Sven Reese, Elke Rauch

**Affiliations:** 1 Department of Veterinary Sciences, Chair of Animal Welfare, Ethology, Animal Hygiene and Animal Husbandry, Faculty of Veterinary Medicine, LMU Munich, Veterinaerstrasse 13/R, 80539 Munich, Germany; 2 Christiane Keppler, Gallicon, Geflügelberatung, Burgstraße 24, 34593 Knüllwald Wallenstein, Germany; 3 Aviagen EPI, Elmpterweg 47, 6042 KJ Roermond, Netherlands; 4 Brüterei Süd ZN of the BWE-Brüterei Weser-Ems GmbH & Co. KG, Peter-Henlein-Strasse 1, 93128 Regenstauf, Germany; 5 Bayerische Landesanstalt für Landwirtschaft, Lehr-, Versuchs- und Fachzentrum für Geflügel- und Kleintierhaltung, Mainbernheimer Straße 101, 97318 Kitzingen, Germany; 6 Department of Veterinary Sciences, Chair of Anatomy, Histology and Embryology, Faculty of Veterinary Medicine, LMU Munich, Veterinaerstrasse 13, 80539 Munich, Germany

**Keywords:** broiler genotype, slow growth, ranger, gait score, welfare indicator

## Abstract

For broiler genotypes to be merchandized under the animal welfare label of the German Animal Welfare Federation, several animal-based welfare indicators with upper limits are listed in a criteria catalog. We compared the prevalence of animal-based welfare indicators in 4 slow-growing broiler genotypes [Ranger Classic (**RC**), Ranger Gold (**RG**), Rowan Ranger (**RoR**), and Rambler Ranger (**RaR**)] in terms of potential approval of these genotypes for a German animal welfare label program. Chicks were housed in 16 floor pens, of which 8 had access to a winter garden. With 4 replications of each genotype, animal-based welfare indicators were assessed in 160 broilers (10 broilers per pen) on fattening days (**FD**) 36 and 44. The body weight of the 4 broiler genotypes differed on both examination days in decreasing order for RC, RG, RoR, and RaR (*P* < 0.001). The walking ability was within the scope of the animal welfare label in all genotypes; it was better in genotypes with a lower mean body weight and correlated positively with the body weight in RG, RoR, RaR, and in the pooled data of the 4 genotypes. Hock burns were only observed at a low severity score, with male broilers being affected more often than female broilers. A positive correlation of the development of hock burn with the weight of the broilers was observed on FD 44 when data of all genotypes were pooled. The footpads of all examined broilers were without lesions at both examinations. Skin scratches were observed in all genotypes at both examinations, and RC broilers differed on FD 36 from the other 3 genotypes by showing a higher prevalence of more severe scratches. Broilers of pens with access to a winter garden were affected by skin scratches more often than broilers without. With the exception of weight gain in 2 genotypes, the investigated indicators showed that all genotypes met the requirements of the animal welfare label.

## INTRODUCTION

Several label programs exist for the rearing of broiler chickens to meet higher welfare standards in Germany and other European countries (German Animal Welfare Federation, 2017; Dierenbescherming, 2018; RSPCA, 2018). In addition to reduced stocking densities, alternative genotypes have been suggested for the use in welfare labels and have been studied regarding productivity or meat quality (Damme et al., 2015) and compliance with indicators of animal welfare and health (Rauch et al., 2017).

In order to define the term “slow growth” of broilers, several regulations should be considered. Commission Regulation (EC) No. 889/2008 lays down detailed rules for the implementation of Council Regulation No. 834/2007 on organic production and labeling of organic products with regard to organic production, labeling, and control. Article 12 of the regulation specifies the minimum age of broilers at slaughter to prevent the use of intensive rearing methods in organic farming. It stipulates that for organic poultry production, slow-growing poultry strains shall be used or that otherwise the age at slaughter should not exceed 81 d. The working group of the federal states on organic farming of Germany stated that slow-growing broilers are those strains whose growth rate is at most 80% of the daily growth rate of the strains bred for top efficiency. The data basis for this calculation is the yearbook for poultry with respective published evaluation results for conventional broilers on-farm (Federal Office for Agriculture and Food, Working Group of the Federal States on Organic Farming, 2009). In the current yearbook for poultry (Damme, 2017), the average daily weight gain of conventional broiler strains is 61.5 or 63.4 g/day for the year 2016, depending on the federal state. Based on the above 80% daily growth rate as definition for “slow growth”, these values allow the interpretation that “slow-growing” is currently defined as a growth rate of at maximum 49.2 to 50.7 g/d. This corresponds to 80% of the current daily weight gain of conventional broiler strains.

The German Animal Welfare Federation, a private association, established an animal welfare label. For broilers, a label program was established in cooperation with scientific, agricultural, trading and processing stakeholders; it includes a 2-staged label with an entry and a premium stage (German Animal Welfare Federation, 2017). The entry stage provides an improvement of animal welfare compared with governmental requirements. This is realized for example by reduced stocking densities, provision of environmental enrichment and a winter garden or the use of alternative genotypes. For the rearing of broilers in the premium stage, the broilers additionally must have access to an outdoor run, a required housing condition in organic farming. By the end of 2018, 60% of all broilers in Germany are supposed to be reared under any kind of welfare label, thus doubling this percentage from currently 30% (PHW Group, 2017). Currently in Germany, approximately 160 000 broilers per week are slaughtered according to the standards of the entry level of the animal welfare label of the German Animal Welfare Federation (PHW Group, 2017). All farmers who participate in this label program are certified and audited on a regular basis by an independent certification authority on compliance with the requirements of the label (German Animal Welfare Federation, 2016). The results of the initial audit determine the frequency of following audits.

In the list of criteria for livestock-appropriate housing and treatment of broilers of the animal welfare label requirements concerning the growth and genotype are mentioned. Broilers of an “extensive” to “middle-extensive” genotype with a confirmed slow growth and an average daily weight gain of maximum 45 g/d are mandatory. Broiler strains not exceeding this limit may be accepted for the animal welfare label (German Animal Welfare Federation, 2017). Only broiler strains approved by the German Animal Welfare Federation are allowed to be used in the label. For the approval of a new genotype, an application must be submitted to the breeder organization program (German Animal Welfare Federation, 2017). The breeder or applicant has to provide data that confirm that the average daily weight gain within 56 fattening days (**FD**) does not exceed 45 g/day. The German Animal Welfare Federation then decides on the approval and might demand more data concerning the weight development and welfare of the genotype, including an assessment of animal welfare indicators. The genotype “Ranger Gold” examined in this study complies with this regulation and is officially approved for the animal welfare label, the genotypes Ranger Gold, Rambler Ranger, and Rowan Ranger for the Better Life label, a welfare label in the Netherlands and the genotypes Ranger Classic, Ranger Gold, and Rambler Ranger for the RSPCA Assured in the UK (Aviagen, 2018a).

Several authors concluded that slow-growing broiler strains perform better with reference to health issues than conventional strains. Growth rate, age and live weight are important factors influencing the gait score and development of lameness (Kestin et al., 2001). Fanatico et al. (2008) found nearly no gait score alterations, a lower prevalence of tibial dyschondroplasia and lower mortality rates in slow-growing broilers strains when compared with fast-growing broilers. Furthermore, Kjaer et al. (2006) did not find footpad dermatitis (**FPD**) and hock burn in slow-growing broilers, whereas fast-growing broiler strains showed first signs of FPD and hock burn in week 2. Bessei (2006) defined the growth rate as main issue for leg disorders and suggested that also genetic traits, nutrition, and diseases should be considered. Also, Keppler et al. (2010) found an increase of lameness, FPD, hock burn, poor plumage condition, and injuries in broilers with higher daily weight gain and higher body weight. Based on these published studies, the requirement of low daily weight gain seems reasonable considering animal welfare. Results from these studies furthermore suggest that the assessment of growth rate and weight gain can be a simple and informative indicator of animal welfare.

However, in some studies, slow-growing broilers performed poorly compared with their fast-growing conspecifics in terms of animal-based welfare indicators. Allain et al. (2009) observed more breast blisters but fewer footpad lesions and Bokkers and Koene (2003) more deformed keel bones in slower growing broilers. In the study of Bokkers and Koene (2003), all broilers (slow- and fast-growing) were supplied with perches, but the fast-growing broilers did not use the perches as frequently as their slow-growing counterparts, which might explain the higher incidence of deformed keel bones in the slower growing broilers. Keppler et al. (2009), on the other hand, assumed that the broad breast of heavier strains prevents lesions because the keel bone is less protruding.

To assess the welfare of broilers, animal-based welfare indicators should be used. Animal- based indicators form a direct measure of animal welfare and represent characteristic traits with respect to animal health and behavior (Zapf et al., 2015). Butterworth et al. (2016) reported that in decreasing order, FPD, dead on arrival, total rejections, ascites, cumulative daily mortality, joint lesions, hock burn, breast lesions, emaciation, wing fractures, cellulites, respiratory disease, and scratches are commonly used as animal-based welfare indicators by competent authorities in the member states of the European Union. The German Animal Welfare Federation presented a list of animal-based welfare indicators for broilers, with limits not to be exceeded in order to be accepted for the animal welfare label (German Animal Welfare Federation, 2017). The threshold values for the indicators are based on the Welfare Quality® assessment protocol for poultry (Welfare Quality®, 2009) as well as the suggestions for implementation of the German Order on the Protection of Animals and the Keeping of Production Animals (German Animal Welfare Federation, 2017). Amongst others, welfare indicators included in the list are gait score, assessed on the farm, as well as FPD and hock burn assessed at slaughter. Up to now, skin scratches are not mentioned in the list of criteria. For the earlier mentioned approval of a new genotype in the welfare label, further animal-based welfare indicators can be used additionally to the average daily weight gain during the process of approval.

The aim of this study was to assess and compare the prevalence of animal-based welfare indicators in 4 slow-growing broiler genotypes. Furthermore, we wanted to examine if correlations existed between these indicators and if the access to a winter garden had an effect on the prevalence of the indicators. Based on the results, we evaluated if the examined indicators are suitable for use in an animal welfare label program and if the, within the label program mandatory winter garden, has an effect. To our knowledge, the comparison of the 4 examined broiler genotypes in terms of animal welfare indicators, i.e., the main goal of this study, has not yet been published. It is relevant to the poultry sector, as these genotypes are used for rearing according to animal welfare labels throughout Europe.

## ANIMALS, MATERIALS, AND METHODS

### Animals and Housing

**Figure 1.**
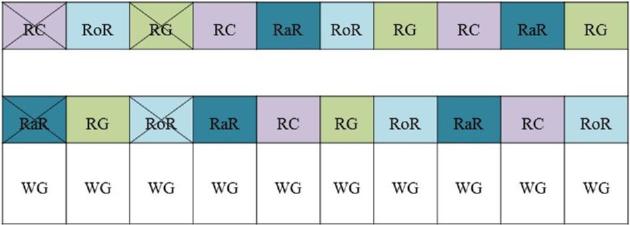
Distribution of the broiler genotypes Ranger Classic (RC), Ranger Gold (RG), Rowan Ranger (RoR), and Rambler Ranger (RaR) in the pens. Crossed pens are excluded from analysis. WG = winter garden.

Broiler chicks (*n* = 2850) were housed in a barn of the Bavarian State Research Center for Agriculture at the Institute for Poultry Education and Applied Research in Kitzingen, Germany, on February 8th, 2017. They had hatched at and were delivered by Brüterei Süd ZN of the BWE-Brüterei Weser-Ems GmbH & Co. KG, Germany. Non-sexed chicks of the genotype Ranger Classic (702 chicks), Ranger Gold (702), Rowan Ranger (723), and Rambler Ranger (723) were housed in floor pens according to the scheme presented in Figure 1. The indoor pens were separated by solid boards preventing visual contact between birds of adjacent pens. Pens in the winter garden were separated by solid boards of approximately 30 cm height with wire mesh above; thus, the birds in adjacent pens could see each other. The length of the fattening period and time of slaughter was determined by the target weight of 1.89 kg, which was reached approximately on FD 40 by Ranger Classic and Ranger Gold and a few days later by Rowan Ranger and Rambler Ranger. Slaughter of all genotypes took place after the target weight was reached by the last genotype (Rambler Ranger) on FD 44. Of each genotype, 2 (Rowan Ranger, Rambler Ranger) or 3 (Ranger Classic, Ranger Gold) replications were housed in pens without access to a winter garden (target stocking density: 25 kg/m^2^, 13.2 broilers per m^2^; size of pen: 10 m^2^) and 2 (Ranger Classic, Ranger Gold) or 3 (Rowan Ranger, Rambler Ranger) replications were housed in pens with access to a winter garden (target stocking density including winter garden: 29 kg/m^2^, related to the inside area, 15.3 broilers per m^2^; size of pen: 10 m^2^; size of winter garden: 5 m^2^ per pen). These stocking densities comply with the entry level of the German animal welfare label (German Animal Welfare Federation, 2017). Access to the winter garden was given from FD 29 onwards, throughout the light phase. For the data analysis, 4 pens were excluded from the statistical analysis to ensure a balanced sampling of pens with and without a winter garden (Figure 1). Due to technical reasons at the research facility, environmental enrichment such as perches, straw bales, or pecking blocks, as required by the animal welfare label, was not supplied. Thus, the broilers were not labeled as broilers of the animal welfare label.

The litter material used was straw granule (1 kg/m^2^). The pens were supplied with feeders, nipple drinkers (including cups) and LED lighting. Conventional standard diet (MEGA Tierernährung GmbH & Co. KG, Visbek-Rechterfeld, Germany) was equally provided ad libitum for all genotypes. The following feed types were used: “Starter” (FD 1 to 8; each genotype 0.15 tons), “Grower I” (FD 9 to 21; each genotype 0.75 tons), “Grower II” (FD 22 to 39; each genotype 2.50 tons), and “Finisher” (FD 40 until slaughter; Ranger Classic and Ranger Gold 0.75 tons, Rowan Ranger and Rambler Ranger 1.25 tons). Starter and Grower Feeds I and II contained a coccidiostat. The barn was supplied with a gas burner and a heat exchanging device and spray cooling to maintain an optimum temperature. The broilers were vaccinated with IB via spray vaccination in the hatchery (1st day of life), ND/IB and Vit. AD3E (12th day of life) and IB and Vit. AD3E (18th day of life) via drinking water. Lighting was performed with 23 h of light and 1 h of darkness from day 1 to 3, 18 h of light and 6 h of darkness from day 4 until 3 days before slaughter. 3 d before slaughter, a lighting program of 23 h of light and 1 h of darkness was performed.

### Methods of Assessment

On FD 36 and FD 44, the animal-based welfare indicators gait score, soiling of the plumage, FPD, hock burn, and skin scratches, as shown in Table 1, were assessed in 160 broilers. Gait score, FPD, and hock burn were assessed according to a modified scheme of the Welfare Quality® assessment protocol for poultry (Welfare Quality®, 2009). Profound lesions of FPD and hock burn were defined as areas where the scales of the footpad or the hock were not adjoined and erosions or ulcerations were present. Ten broilers (5 male and 5 female) of each of the 4 replicate pens were examined on FD 36 and FD 44, resulting in 40 assessed broilers of each genotype at each examination. We selected FD 36 and FD 44 for the examination because on these FD, the target weight was reached by 2 (FD 36), respectively all (FD 44) of the genotypes. Furthermore, in the audits of the label program, the gait score is examined within the last fattening week and FPD and hock burn at time of slaughter (German Animal Welfare Federation, 2017). First, the gait score of the broilers was assessed by 1 assessor without touching or handling the animal before scoring. Then, the animals were weighed and the other welfare indicators were evaluated by 2 further assessors. The inter-observer reliability of these 2 assessors was evaluated by the use of the PABAK (prevalence-adjusted and bias-adjusted kappa) calculation (Byrt et al., 1993) for soiling of the plumage, hock burn, FPD, and skin scratches in 200 broilers. According to Gunnarsson et al. (2000), if more than 2 categories are evaluated, the calculation is performed as follows: PABAK = (kp0 − 1)/(k − 1), where k represents the number of assessed categories (degree of injury) and p0 the proportion of matching between the observers.

**Table 1.** Assessed animal-based welfare indicators with respective score used.

Scientific Score	Soiling of plumage	Skin scratches	Gait score^a^	Footpad dermatitis^a^	Score in welfare label for gait score and footpad dermatitis^b^	Hock burn^a^	Score in welfare label for hock burn^b^
0	Plumage clean	None	Normal gait, dexterous and agile, chicken-typical gait	No lesion	0	No lesion	0
1	Mild soiling (only ventral)	Mild scratches (superficial)	Slight abnormality, but difficult to define	Superficial^c^ lesion, small area (≤0.5 cm)	0	Redness	0
2	Moderate soiling (ventral and dorsal)	Moderate scratches (dermis penetrated, 1-sided)	Slight lameness, but affected leg is not identifiable	Superficial^c^ lesion, large area (>0.5 cm) or profound^d^ lesion, small area (≤0.5 cm)	1	Superficial^c^ lesion, small area (≤.5 cm)	0
3	Severe soiling (complete body)	Severe scratches (dermis penetrated, 2-sided)	Distinct lameness, affected leg is identifiable	Profound^d^ lesion, large area (>0.5 cm)	1	Superficial^c^ lesion, large area (>0.5 cm) or profound^d^ lesion, small area (≤0.5 cm)	1
4	n/a	n/a	Broiler walks only a few steps	Profound^d^ lesion, whole footpad including toe	1	Profound^d^ lesion, large area (>0.5 cm)	1
5	n/a	n/a	Incapable of walking	n/a	1	Profound^d^ lesion, whole hock	1

n/a = not applicable.

^a^Adjusted according to Welfare Quality® (2009).

^b^Maximum of 10% of the broilers with gait score 1, hock burn score 1 or 20% with footpad dermatitis score 1 in the animal welfare label.

^c^Lesions that are not profound, no erosion, no ulceration.

^d^Profound lesions of footpad dermatitis and hock burn were defined as areas where the scales of the footpad or the hock were not adjoined and erosions or ulcerations were present.

The litter quality was scored weekly, starting on FD 7, according to a 5-staged scoring system of the Welfare Quality® assessment protocol for poultry, with Score 0 denoting “completely dry and flaky litter, moves easily with the foot; Score 1 = dry but not easy to move with foot; Score 2 = leaves imprint of foot and will form a ball if compacted, but ball does not stay together; Score 3 = sticks to boots and sticks readily in a ball if compacted; and Score 4 = sticks to boots once the cap or compacted crust is broken” (Welfare Quality®, 2009).

### Statistical Methods

Data regarding animal health (gait score, soiling of plumage, FPD, hock burn, skin scratches) were recorded as coherent data for each broiler separately. Analysis of data was performed with IBM SPSS Statistics 24.0 software (IBM Deutschland GmbH, Ehningen, Germany) and MedCalc Statistical Software version 17.8.2 (MedCalc Software bvba, Ostend, Belgium; https://www.medcalc.org; 2017). The comparative analysis of the genotypes and the weight of the broilers were performed with a univariate analysis of variance. For all further parameters, non-parametric tests were used for the statistical evaluation. For the indicators gait score, soiling of plumage, FPD, hock burn, and skin scratches, a Kruskal–Wallis test with following post-hoc analysis with a paired comparison using a Dunn–Bonferroni test and Bonferroni correction was performed. Before analysis, Kolmogorov–Smirnov tests confirmed that the data were not normally distributed. Correlations were analyzed using the Spearman correlation coefficient. The strength of a relationship can be described as follows (Rea and Parker, 2014): 0.00 to ≤0.10 = negligible, 0.11 to ≤0.20 = weak, 0.21 to ≤0.40 = moderate, 0.41 to ≤0.60 = relatively strong, 0.61 to ≤0.80 = strong and 0.81 to ≤1.00 = very strong. Differences between pens with or without access to a winter garden, between sexes, and between ages were individually assessed using a Chi-Squared test. Additionally, a multifactorial analysis was performed. A generalized linear model for the dependent variables body weight, gait score, soiling of plumage, hock burn, and skin scratches was used. The factors genotype, winter garden, gait score, and sex as well as the interactions of the factors genotype and body weight and sex and body weight were included as predictors in the models for each indicator. The body weight was included as covariate.

To assess possible differences regarding the confounding variable “winter garden,” the first pens of each genotype with winter garden or without that were overrepresented were eliminated from the data sheet to calculate with a balanced number of sample size (1 pen each of Rambler Ranger and Rowan Ranger with winter garden and 1 pen each of Ranger Gold and Ranger Classic without winter garden; Figure 1). Results were regarded as significant if *P* ≤ 0.05.

## RESULTS AND DISCUSSION

The PABAK inter-observer reliability test showed high inter-observer reliability for the animal welfare indicators: soiling of plumage (PABAK = 0.95), hock burn (PABAK = 0.93), FPD (PABAK = 0.99), and skin scratches (PABAK = 0.96). These results indicate an almost perfect reliability as stated by Landis and Koch (1977), who proposed a range of a Kappa Value 0.81 to 1 as almost perfect reliability.

### Weight Development

All of the examined broilers showed on both examination days (FD 36 and FD 44) an age-appropriate development and good nutritional state (no “undersized” broilers). The body weight of male broilers was significantly higher than that of female broilers at both examinations, and effects of sex and genotype on the body weight were observed in the multifactorial analysis (FD 36: *P* < 0.001; FD 44: *P* < 0.001). Broilers of the genotype Ranger Classic had the highest average body weights at both examinations (FD 36: 2092 g; FD 44: 2660 g; Tables 2 and 3). Ranger Gold broilers weighed more on FD 36 and FD 44 than Rowan Ranger and Rambler Ranger. Broilers of the genotype Rambler Ranger had a lower average body weight than the other 3 genotypes. The average body weights differed between the 4 genotypes on both examination days (*P* < 0.001; Tables 2 and 3). The performance objectives of the breeder Aviagen for the genotype Ranger Classic define a target weight of 1604 g for FD 36 and 2131 g for FD 44 (Aviagen, 2018b). The higher average body weights of this genotype in our study could be explained by the experimental conditions and small pens in which the broilers were housed. Under these standardized conditions, the broilers might have had a higher feed intake than they would have had under practical field conditions. Rowan Ranger broilers are defined to have a target weight of 1283 g on FD 36 and 1705 g on FD 44 and weighed slightly more in our examinations than expected, but they exceeded the target weight less than the Ranger Classic broilers (Aviagen, 2018c). The average daily weight gain was 61.0 g for Ranger Classic, 54.3 g for Ranger Gold, 44.1 g for Rowan Ranger, and 37.5 g for Rambler Ranger. Thus, the genotypes Ranger Classic and Ranger Gold exceeded the recommended limit of the German Animal Welfare Federation (2017). However, this might be due to the standardized fattening conditions and small housing groups under pen trial conditions and are not to be expected under field conditions. First observations under field conditions show a lower weight gain than in the presented trial and will be published as a case study.

**Table 2.** Distribution of assessed animal-based welfare indicators (in percent) in the 4 genotypes, including significant differences, on FD 36. Different letters indicate statistical difference (*P* ≤ 0.05).

			Genotype							
			Ranger		Ranger		Rowan		Rambler	
Indicator	Statistics	Score	Classic		Gold		Ranger		Ranger	
Weight (g)	*F* = 75.684		2092 ± 314		1814 ± 224		1529 ± 283		1300 ± 148	
ANOVA	*P* < 0.001		a		b		c		d	
			%	*n*	%	*n*	%	*n*	%	*n*
Gait score		0	7.5	3	10.0	4	47.5	19	77.5	31
		1	92.5	37	85.0	34	47.5	19	22.5	9
		2	0.0	0	0.0	0	2.5	1	0.0	0
		3	0.0	0	0.0	0	0.0	0	0.0	0
		4	0.0	0	5.0	2	2.5	1	0.0	0
		5	0.0	0	0.0	0	0.0	0	0.0	0
Kruskal–Wallis test	*P* < 0.001		a		a		b		c	
Soiling of plumage		0	80.0	32	85.0	34	97.5	39	100.0	40
		1	20.0	8	15.0	6	2.5	1	0.0	0
		2	0.0	0	0.0	0	0.0	0	0.0	0
		3	0.0	0	0.0	0	0.0	0	0.0	0
Kruskal–Wallis test	*P* < 0.05		a		ab		b		b	
Hock burn		0	87.5	35	95.0	38	80.0	32	92.5	37
		1	2.5	1	2.5	1	0.0	0	0.0	0
		2	7.5	3	2.5	1	20.0	8	7.5	3
		3	2.5	1	0.0	0	0.0	0	0.0	0
		4	0.0	0	0.0	0	0.0	0	0.0	0
		5	0.0	0	0.0	0	0.0	0	0.0	0
Kruskal–Wallis test	*P* = 0.144									
Footpad dermatitis		0	100.0	40	100.0	40	100.0	40	100.0	40
		1	0.0	0	0.0	0	0.0	0	0.0	0
		2	0.0	0	0.0	0	0.0	0	0.0	0
		3	0.0	0	0.0	0	0.0	0	0.0	0
		4	0.0	0	0.0	0	0.0	0	0.0	0
Skin scratches		0	27.5	11	60.0	24	75.0	30	85.0	34
		1	60.0	24	30.0	12	25.0	10	10.0	4
		2	12.5	5	10.0	4	0.0	0	5.0	2
		3	0.0	0	0.0	0	0.0	0	0.0	0
Kruskal–Wallis test	*P* < 0.001		a		b		bc		c	

**Table 3.** Distribution of assessed animal-based welfare indicators (in percent) in the 4 genotypes, including significant differences, on FD 44. Different letters indicate statistical difference (*P* ≤ 0.05).

			Genotype							
			Ranger		Ranger		Rowan		Rambler	
Indicator	Statistics	Score	Classic		Gold		Ranger		Ranger	
weight (g)	*F* = 72.401		2660 ± 410		2342 ± 354		1902 ± 330		1645 ± 221	
ANOVA	*P* < 0.001		a		b		c		d	
			%	*n*	%	*n*	%	*n*	%	*n*
Gait score		0	5.0	2	15.0	6	25.0	10	72.5	29
		1	95.0	38	82.5	33	75.0	30	27.5	11
		2	0.0	0	2.5	1	0.0	0	0.0	0
		3	0.0	0	0.0	0	0.0	0	0.0	0
		4	0.0	0	0.0	0	0.0	0	0.0	0
		5	0.0	0	0.0	0	0.0	0	0.0	0
Kruskal–Wallis test	*P* < 0.001		a		a		a		b	
Soiling of plumage		0	65.0	26	87.5	35	97.5	39	100.0	40
		1	35.0	14	12.5	5	2.5	1	0.0	0
		2	0.0	0	0.0	0	0.0	0	0.0	0
		3	0.0	0	0.0	0	0.0	0	0.0	0
Kruskal–Wallis test	*P* < 0.001		a		ab		b		b	
Hock burn		0	57.5	23	77.5	31	80.0	32	87.5	35
		1	5.0	2	7.5	3	5.0	2	0.0	0
		2	30.0	12	15.0	6	15.0	6	12.5	5
		3	7.5	3	0.0	0	0.0	0	0.0	0
		4	0.0	0	0.0	0	0.0	0	0.0	0
		5	0.0	0	0.0	0	0.0	0	0.0	0
Kruskal–Wallis test	*P* = 0.009		a		ab		ab		b	
Footpad dermatitis		0	100.0	40	100.0	40	100.0	40	100.0	40
		1	0.0	0	0.0	0	0.0	0	0.0	0
		2	0.0	0	0.0	0	0.0	0	0.0	0
		3	0.0	0	0.0	0	0.0	0	0.0	0
		4	0.0	0	0.0	0	0.0	0	0.0	0
Skin scratches		0	55.0	22	55.0	22	70.0	28	85.0	34
		1	37.5	15	45.0	18	25.0	10	12.5	5
		2	7.5	3	0.0	0	5.0	2	2.5	1
		3	0.0	0	0.0	0	0.0	0	0.0	0
Kruskal–Wallis test	*P* = 0.016		a		a		ab		b	

### Gait Score

Regarding the gait score, 2 broilers each of the genotypes Ranger Gold (5%) and Rowan Ranger (5%) showed a gait score of 2 or worse on FD 36 (Table 2). On FD 44, only 1 broiler of the genotype Ranger Gold was assessed with a gait score of 2 (Table 3). The genotypes Rowan Ranger and Rambler Ranger (*P* < 0.0001) showed a better walking ability on FD 36 than Ranger Classic and Ranger Gold (Table 2). On both FD, broilers of the genotype Rambler Ranger were assessed with the best walking ability (*P* < 0.001). It should be emphasized that the gait scores of all genotypes fell within the scope of the animal welfare label issued by the German Animal Welfare Federation (German Animal Welfare Federation, 2017), and differences observed were mostly in the range of “normal gait, dexterous and agile, chicken-typical gait” (Score 0), and “slight abnormality, but difficult to define” (Score 1). According to the catalog of criteria, at most 10% of the assessed broilers may show a gait score of 2 or above for acceptance in the label (German Animal Welfare Federation, 2017). Within the fattening period, no broilers had to be culled due to leg weakness.

Our assessment of gait scores on FD 36 and FD 44 revealed a positive correlation with the body weight in 3 genotypes (Ranger Gold, Rowan Ranger, and Rambler Ranger), and this correlation was strong when the data from all genotypes were pooled (Tables 4 and 5). Overall, the lower the body weight of assessed broilers, the lower was their gait score and the better was their walking ability. This finding was confirmed in the multifactorial analysis, in which the body weight had a significant effect on the gait score on FD 36 (*P* < 0.001) and FD 44 (*P* = 0.001; Table 6). Kestin et al. (2001) showed that slow-growing broilers have a better ability to walk and that the body weight and growth rate of the broilers are important factors for the development of lameness. Keppler et al. (2010) supported this finding with their examinations of slow-growing broiler breeds for organic production. In their study, percentages of broilers without lameness decreased with higher daily weight gain and higher body weight (Keppler et al., 2010). Furthermore, Kestin et al. (2001) observed a higher regression coefficient between body weight and gait score in younger than in older broilers. The authors concluded that younger broilers are more sensitive to differences in live weight than older birds. These findings correspond to the results of our study. Keppler et al. (2009) and Rauch et al. (2017) also observed a relationship between the ability to walk and the body weight of the broilers.

**Table 4.** Correlations (Spearman rho) of the weight and gait score with other assessed parameters such as soiling of plumage, hock burn, and skin scratches, separated into the 4 genotypes on FD 36.

		Genotype				
Analysis	Statistics	Ranger Classic	Ranger Gold	Rowan Ranger	Rambler Ranger ^a^	Total
Correlation (Spearman rho) body weight with:	*n*	40	40	40	40	160
Hock burn	*r*	0.352	0.165	0.022	0.341	0.093
	*P*	0.026	0.309	0.894	0.031	0.244
Soiling of plumage	*r*	−0.133	−0.112	0.229	-	0.193
	*P*	0.414	0.491	0.155	-	0.015
Skin scratches	*r*	−0.329	0.159	0.035	−0.003	0.311
	*P*	0.038	0.328	0.830	0.985	<0.001
Correlation (Spearman rho) gait score with:	*n*	40	40	40	40	160
Body weight	*r*	0.292	0.351	0.735	0.573	0.725
	*P*	0.068	0.026	<0.001	<0.001	<0.001
Soiling of plumage	*r*	−0.332	0.059	0.133	-	0.132
	*P*	0.036	0.719	0.413	-	0.095
Hock burn	*r*	−0.221	0.032	−0.049	−0.153	−0.037
	*P*	0.170	0.844	0.765	0.345	0.646

^a^Dashes indicate missing correlation due to non-existent soiling of plumage.

**Table 5.** Correlations (Spearman rho) of the weight and gait score with other assessed parameters such as soiling of plumage, hock burn, and skin scratches, separated into the 4 genotypes on FD 44.

		Genotype				
Analysis	Statistics	Ranger Classic	Ranger Gold	Rowan Ranger	Rambler Ranger ^a^	Total
Correlation (Spearman rho) body weight with:	*n*	40	40	40	40	160
Hock burn	*r*	0.155	0.376	0.191	0.475	0.345
	*P*	0.341	0.017	0.238	0.002	<0.001
Soiling of plumage	*r*	−0.045	0.134	0.062	–	0.315
	*P*	0.781	0.409	0.702	–	<0.001
Skin scratches	*r*	−0.002	0.226	0.149	−0.043	0.259
	*P*	0.990	0.160	0.359	0.794	0.001
Correlation (Spearman rho) gait score with:	*n*	40	40	40	40	160
Body weight	*r*	0.050	0.451	0.545	0.361	0.606
	*P*	0.761	0.003	<0.001	0.022	<0.001
Soiling of plumage	*r*	−0.072	0.124	0.092	–	0.196
	*P*	0.658	0.446	0.570	–	0.013
Hock burn	*r*	−0.112	0.318	−0.165	0.275	0.177
	*P*	0.490	0.045	0.308	0.086	0.025

^a^Dashes indicate missing correlation due to non-existent soiling of plumage.

**Table 6.** *P*-Values of the multifactorial analysis using the generalized linear model for the dependent variables body weight, gait score, soiling of plumage, hock burn, and skin scratches; FD = Fattening Day.

	Dependent variables				
	Body weight	Gait score	Soiling of plumage	Hock burn	Skin scratches
FD 36					
*Predictors*					
Genotype	<0.001	0.072	0.740	0.388	0.146
Winter garden	0.448	0.127	0.684	0.151	0.001
Sex	<0.001	0.058	0.535	0.570	0.121
Gait score	–	–	0.993	0.611	0.443
Interaction genotype*body weight	–	0.056	0.755	0.781	0.104
Interaction sex*body weight	–	0.087	0.425	0.471	0.178
*Covariate*					
Body weight	–	<0.001	1.000	0.091	0.860
FD 44					
*Predictors*					
Genotype	<0.001	0.477	0.701	0.037	0.354
Winter garden	0.150	0.002	0.996	0.741	0.036
Sex	<0.001	0.339	0.618	0.262	0.117
Gait score	–	–	0.959	0.244	0.286
Interaction genotype*body weight	–	0.491	0.824	0.194	0.220
Interaction sex*body weight	–	0.292	0.696	0.048	0.257
*Covariate*					
Body weight	–	<0.001	1.000	0.030	0.033

In the pooled dataset of all genotypes, we observed a weak positive correlation between the gait score and the soiling of the plumage on FD 44 (*r* = 0.196, *P* = 0.013; Table 5). On FD 36, we observed no correlation between the ability to walk and the development of contact dermatitis of the hock (hock burn) (Table 4), whereas on FD 44, we found a weak correlation, both for the evaluation of the pooled data (*r* = 0.177, *P* = 0.025) and for the genotype Ranger Gold (*r* = 0.318, *P* = 0.045) (Table 5). It should be considered however, that hock burn was only observed at a low severity score. Rauch et al. (2017) also observed a weak correlation between the gait score and the development of hock burn in a comparative study of 4 slow-growing broiler genotypes. Kristensen et al. (2006) observed that heavier broilers have an increased risk of high gait and hock burn scores and found a correlation between the 2 parameters (gait and hock burn). However, they concluded that the direction of the correlation could not be specified by their investigation and it should be examined whether lame birds spend more time sitting and thus have a higher risk of contact dermatitis of the hock or if contact dermatitis of the hock causes the lameness. In our study, female broilers showed a better walking ability than male broilers on both assessment days (FD 36: *P* < 0.001; FD 44: *P* = 0.042), which might be linked to the lower body weight of female broilers. The effect of sex was not confirmed in the multifactorial analysis. Observations of female broilers having a better walking ability than male broilers were also made by Sørensen et al. (2000).

### Soiling of Plumage

In the heavier genotypes Ranger Classic and Ranger Gold, we observed more broilers with soiled plumage on both examination days (FD 36 and FD 44) compared with the lighter genotypes Rowan Ranger and Rambler Ranger (Tables 2 and 3). All (100%) of the assessed Rambler Ranger broilers showed clean plumage without soiling and differed in this aspect (as well as the genotype Rowan Ranger) from the genotype Ranger Classic on FD 36 and FD 44 (*P* < 0.05) (Tables 2 and 3). In the multifactorial analysis, the effect of genotype or body weight was not observed (Table 6); however, in a following uni-factorial analysis, an effect of genotype (FD 44: *P* = 0.01) and body weight (FD 36: *P* = 0.032; FD 44: *P* < 0.001) was observed. Westermaier (2015) observed in her study that more of the fast-growing Ross 308 broilers showed soiling of plumage compared with the slower growing broiler strain Cobb Sasso. Rauch et al. (2017) also found a correlation between the body weight of the broilers and the soiling of plumage in a comparison of 4 slow-growing broiler genotypes.

### Hock Burn

Contact dermatitis on the hock (hock burn) was only observed at a low severity score (maximum observed score = 3) and only in a few individual broilers (Tables 2 and 3). Hock burn score 3 (superficial lesion, large area) was only observed in broilers of the genotype Ranger Classic (FD 36: 2.5%; FD 44: 7.5%). On FD 44, the genotype Ranger Classic differed from the genotype Rambler Ranger in terms of the development of hock burn (*P* = 0.009) (Table 3). Thus, concerning hock burn, all examined broilers of all genotypes complied with the demands of the catalog of criteria for the animal welfare label issued by the German Animal Welfare Federation. This catalog defines that a hock burn of score 3 (superficial lesion, large area) or worse should not occur in more than 10% of assessed broilers (German Animal Welfare Federation, 2017).

Similar to an observation by McKeegan (2010), hock burns were more frequently observed in broilers of older age. Even though several authors found a relationship between the weight of the broilers and the development of hock burn, this was only the case for 2 genotypes on FD 36 in our study (Ranger Classic, Rambler Ranger; Table 4). On FD 44, we found a correlation of body weight and hock burn when evaluating the pooled data of all genotypes (*P* < 0.001), and the weight of the broilers of the genotypes Ranger Gold and Rambler Ranger correlated with the presence of hock burn (Table 5) as also observed by other authors (Sørensen et al., 2000; Broom and Reefmann, 2005; Haslam et al., 2007; Keppler et al., 2010; Bergmann et al., 2016; Saraiva et al., 2016; Louton et al., 2018). In our study, male broilers had more hock burn lesions than female broilers (FD 36: *P* = 0.073; FD 44: *P* = 0.023), which was also observed by McKeegan (2010). Because the male broilers had a higher body weight than the female broilers and because the presence of hock burn is correlated with the body weight, this result can be explained by the higher body weight of the male broilers. The multifactorial analysis confirmed the effects of body weight (FD 44: *P* = 0.030), genotype (FD 44: *P* = 0.037), and the interaction of body weight and genotype (FD 44: *P* = 0.048) on the presence of hock burn (Table 6). The effect of sex was not confirmed by the multifactorial model.

### Footpad Dermatitis

The footpads of 100% of the assessed broilers at both examinations (FD 36 and FD 44) were intact and had no lesions (100% with FPD score 0; Tables 2 and 3). The good footpad health of the broilers in our study was likely influenced by the presence of dry litter. The litter quality was very good and dry throughout the entire fattening period; the average score on FD 35 in pens of all genotypes was 1.0, and that on FD 42 was 1.0 in pens of Rambler Ranger and Rowan Ranger, 1.1 in pens of Ranger Classic and 1.2 in pens of Ranger Gold. Several authors stated that FPD is affected by the moisture of the litter (Haslam et al., 2007; Allain et al., 2009; McKeegan, 2010; De Jong et al., 2014).

### Skin Scratches

Skin scratches in broilers are a welfare problem, in particular, because they are often correlated with high mortality rates (Louton et al., 2018). Cellulitis as a possible result of skin scratches was mentioned to be the major reason for condemnation at slaughter (Elfadil et al., 1996). One of the important factors discussed as a cause referring to severity and dimension of skin scratches and consequently cellulitis is the stocking density (Harris et al., 1978; Dozier et al., 2005; Allain et al., 2009). However, body weight and the growth rate of broilers are also mentioned as possible factors influencing skin scratches (Elfadil et al., 1996). At the first examination, on FD 36, 27.5% of the broilers of the genotype Ranger Classic were observed without skin scratches, 60% had superficial scratches without penetration of the dermis, and 12.5% had moderate to severe scratches with penetration of the dermis (Table 2). Moderate and severe scratches with penetration of the dermis were not found in the genotype Rowan Ranger. Broilers of the genotype Ranger Classic differed in the prevalence of skin scratches on FD 36 from all other 3 examined genotypes (*P* < 0.001). On FD 44, we observed fewer skin scratches than on FD 36 in broilers of the genotype Ranger Classic (55% Ranger Classic without skin scratches; Table 3). Fewer skin scratches in older broilers were also observed by Louton et al. (2018). Possibly, older broilers scratch each other less because of their lower activity level as compared with younger broilers (Bokkers and Koene, 2003). We observed no differences regarding skin scratches between the genotypes Ranger Classic, Ranger Gold, and Rowan Ranger on FD 44 (Table 3). Rambler Ranger, the slowest growing broiler line, showed the lowest prevalence of skin scratches.

A correlation of skin scratches with the body weight of the broilers was observed on FD 36 (*r* = 0.311, *P <* 0.001) and FD 44 (*r* = 0.259, *P* = 0.001) when evaluating the pooled data (Tables 4 and 5). The effect of body weight on skin scratches was confirmed by the multifactorial analysis on FD 44 (*P* = 0.033; Table 6). Stocking density, as mentioned by other authors as possible cause, could not be evaluated in our study (Harris et al., 1978; Dozier et al., 2005; Allain et al., 2009). The animal-based welfare indicator “skin scratches” is up to now not mentioned in the catalog of criteria for the animal welfare label issued by the German Animal Welfare Federation (German Animal Welfare Federation, 2017). Due to the welfare aspect of this indicator (Louton et al., 2018), we recommend including this parameter as a criterion for animal welfare in a label program.

### Winter Garden

Pens with and without a winter garden were tested for differences regarding the different welfare indicators. On FD 36 and FD 44, broilers of pens with a winter garden showed more skin scratches than those without access to a winter garden (FD 36: *P* = 0.018; FD 44: *P* = 0.113; both FD: *P* = 0.005). The effect of the winter garden on the prevalence of skin scratches was confirmed in the multifactorial analysis on FD 36 (*P* = 0.001) and FD 44 (*P* = 0.036; Table 6). This finding seems reasonable because the winter garden induces a higher activity level of the broilers in this area (Bergmann et al., 2017) and higher activity levels can possibly lead to more skin damage of the broilers (Weise, 2007). Other factors of the winter garden that might influence skin scratches, such as environmental enrichment and stocking density, should be further investigated. Furthermore, broilers with access to a winter garden showed a better walking ability (gait score lower) on FD 36 (*P* = 0.050) and FD 44 (*P* = 0.009) than broilers without access (both FD: *P* = 0.001). The effect of the winter garden on the gait score was confirmed in the multifactorial analysis on FD 44 (*P =* 0.002). Reiter and Bessei (2009) observed that an increase in locomotive activity may reduce leg disorders. The other welfare indicators were not significantly affected by the variable winter garden.

### Conclusion

Overall, the examined welfare indicators of all genotypes were within the scope of the catalog of criteria for the animal welfare label issued by the German Animal Welfare Federation (German Animal Welfare Federation, 2017), with the exception of weight gain, which was too high in 2 of the examined genotypes. This weight gain might have been the result of the housing condition in small pens and thus a higher food intake than expected under practical conditions. Because the walking ability and the prevalence of hock burn are linked to the weight gain, the average daily weight gain should be monitored and documented weekly by the farm manager, potentially in combination with the prevalence of the mentioned animal welfare indicators. The animal welfare indicators gait score, FPD and hock burn in all 4 genotypes were within the threshold values of the catalog of criteria for the animal welfare label. Skin scratches occur in slow-growing broiler breeds and thus should be incorporated in the audits of the animal welfare label. Because the availability of a winter garden has an effect on the gait score and the prevalence of skin scratches, the use of environmental enrichment should be further evaluated, particularly to reduce skin scratches. However, the results of this study should be interpreted with care, because for the assessment of correlations in the 4 genotypes, a dataset of 40 broilers per genotype on each assessment day seems to be too low for a final evaluation. The results of our study should be the basis for further confirmatory experiments, including practical field conditions.
